# It Is High Time for Personalized Dietary Counseling in Celiac Disease: A Systematic Review and Meta-Analysis on Body Composition

**DOI:** 10.3390/nu13092947

**Published:** 2021-08-25

**Authors:** Zsófia Vereczkei, Nelli Farkas, Péter Hegyi, Marcell Imrei, Mária Földi, Zsolt Szakács, Szabolcs Kiss, Margit Solymár, Rita Nagy, Judit Bajor

**Affiliations:** 1 Institute for Translational Medicine, Medical School, University of Pécs, 7624 Pécs, Hungary; vereczkei47@gmail.com (Z.V.); nelli.farkas@aok.pte.hu (N.F.); marcell.imrei@gmail.com (M.I.); foldimarcsi4@gmail.com (M.F.) szaki92@gmail.com (Z.S.); kissszabolcs1995@gmail.com (S.K.); margit.solymar@aok.pte.hu (M.S.); nagyrita003@gmail.com (R.N.); 2 Department of Sport Nutrition and Hydration, Institute of Emergency Care and Pedagogy of Health, Faculty of Health Sciences, University of Pécs, 7621 Pécs, Hungary; 3 Institute of Bioanalysis, Medical School, University of Pécs, 7624 Pécs, Hungary; 4 Institute for Translational Medicine, Szentágothai Research Centre, Medical School, University of Pécs, 7624 Pécs, Hungary; hegyi2009@gmail.com; 5 Centre for Translational Medicine, Semmelweis University, 1085 Budapest, Hungary; 6 Division of Pancreatic Diseases, Heart and Vascular Center, Semmelweis University, 1085 Budapest, Hungary; 7 Doctoral School of Clinical Medicine, University of Szeged, 6720 Szeged, Hungary; 8 Division of Gastroenterology, First Department of Medicine, Medical School, University of Pécs, 7624 Pécs, Hungary; 9 Heim Pál National Pediatric Institute, 1089 Budapest, Hungary

**Keywords:** celiac disease, gluten-free diet, body composition

## Abstract

The body composition of patients with celiac disease (CD), on which the effects of a gluten-free diet (GFD) are controversial, differs from that of the average population. In this study, we aimed to compare the body composition across CD patients before a GFD, CD patients after a one-year GFD and non-celiac control subjects. A systematic search was conducted using five electronic databases up to 15 July 2021 for studies that reported at least one of the pre-specified outcomes. In meta-analyses, weighted mean differences (WMDs) with 95% confidence intervals (CIs) were calculated. A total of 25 studies were eligible for systematic review, seven of which were included in meta-analysis. During a ≥1-year GFD, fat mass of CD patients, compared to that at baseline, significantly increased (WMD = 4.1 kg, 95% CI = 1.5 to 6.6, three studies). In CD patients after a ≥1-year GFD, compared to non-celiac controls, fat mass (WMD = −5.8 kg, 95% CI = −8.7 to −2.9, three studies) and fat-free mass (WMD = −1.9 kg, 95% CI = −3.0 to −0.7, three studies) were significantly lower. In conclusion, body composition-related parameters of CD patients differ from that of the non-celiac control subjects even after a longstanding GFD.

## 1. Introduction

Celiac disease (CD) is a chronic, immune-mediated systemic disorder induced by gluten proteins in genetically susceptible individuals [1]. The only effective treatment for CD is a strict, lifelong gluten-free diet (GFD), excluding gluten proteins in wheat (gliadins and glutenins), barley (hordein), rye (secalin) and other related grains. CD is one of the most frequent genetically determined disorders, affecting approximately 1% of the world population [2]. The immune-mediated inflammatory reaction can lead to malabsorption and consequent nutrient deficiencies, which can be reversed by a GFD [3].

The diverse clinical presentation of CD includes classical, non-classical, symptomatic, asymptomatic, potential and refractory CD [3]. However, the most significant phenotypes are the classical, non-classical and asymptomatic ones [4]. Formerly, classical CD with malnutrition due to intestinal malabsorption was the most prevalent [5]. Recently, the proportion of non-classical and asymptomatic CD patients with normal or high body weight (BW) already at the time of the diagnosis has been increasing rapidly, which can be attributed, among other things, to increasing disease awareness and accurate and accessible serological testing [6,7,8].

The current guidelines propose no recommendations regarding the need for baseline and follow-up body composition assessment. To assess CD patients’ nutritional status comprehensively and to monitor therapeutic response, body composition-related parameters, such as fat mass (FM) and fat-free mass (FFM), should be evaluated [9]. Several studies suggested that there is an important difference in body composition across (1) untreated CD patients, (2) treated CD patients and (3) non-celiac control subjects. Generally, most classical CD patients not adhering to a GFD are underweight and have lower body mass index (BMI), FM, FFM and bone mass compared to a non-celiac control group [10,11]. After introducing a GFD, the intestinal mucosa heals, the proinflammatory response ceases and the absorption of nutrients is restored [12]. These factors together can cause an increase in BW, BMI, FM, FFM and bone mass, which serves a potential explanation for the difference in body composition observed between treated CD patients and non-celiac controls. However, this response remains undetected in a fraction of the cases [13,14,15]. Lack of complete response can be due to dietary transgressions or failure to achieve mucosal healing, being typical in cases diagnosed late in adulthood. In contrast, evidence suggests that anthropometric parameters of CD patients do not differ from age- and sex-matched control subjects, so that early diagnosis and good dietary adherence can facilitate the restoration of normal body composition [16].

Changes in body composition are not always favorable: An unbalanced GFD can also be responsible for the undesirable changes in both BW and body composition-related parameters. Gluten-free products often have an inappropriate nutritional composition because of high energy density due to high simple carbohydrate and saturated fat content [17]. The increased consumption of these macronutrients together with the improved absorption can lead to unfavorable changes in body composition, mainly a substantial gain in FM and a modest increase in FFM. Thus, the result can be disproportionate body composition and metabolic alterations, including the frequent development of nutrition-related disorders, such as non-alcoholic fatty liver disease (NAFLD) [18]. Moreover, CD patients who are overweight at diagnosis have a higher risk of cardiovascular events and developing metabolic alterations, compared to non-overweight CD patients [8]. In summary, body composition of CD patients can differ from that of non-celiac subjects and data about the effects of a GFD on body composition of CD patients are controversial.

This meta-analysis and systematic review aimed to evaluate the change in body composition of CD patients before introducing a GFD and after at least a one-year GFD. Besides, we aimed to compare these two groups of CD patients to non-celiac control subjects.

## 2. Materials and Methods

This systematic review and meta-analysis is reported in conformity with the Preferred Reporting Items for Systematic Reviews and Meta-Analyses (PRISMA) Statement (Table S1) [19]. The protocol of this study was registered under registration number CRD42021229522 in PROSPERO. It must be declared that there was a deviation from the protocol as we removed the biochemical parameters from the list of the outcomes to preserve the focus of the study.

### 2.1. Data Sources and Search Strategy

A systematic search was conducted using five major literature databases, including MEDLINE (via PubMed), Embase, Cochrane Register of Controlled Trials (CENTRAL), Web of Science and Scopus, from inception to 15 July 2021. We designed a search key, which contains terms associated with CD and body composition and uses the Boolean operators: (“celiac disease” OR “celiac patient*” OR “coeliac disease” OR “coeliac patient*” OR “gluten”) AND ((body composition) OR (“body fat”) OR (“anthropometry”) OR (“body analysis”) OR (“fat mass”) OR (fat percent*) OR (fat proportion) OR (“fat free mass”) OR (“fat free percent*”) OR (“fat free proportion”) OR (“lean mass”) OR (“lean body”) OR (“impedance”) OR (“bia”)). In-built database filters were only applied in the case of Scopus (Article title, Abstract, Keywords). Furthermore, reference lists of the relevant studies were manually screened for any additional studies. We did not contact the authors of the primary studies for further data.

### 2.2. Selection and Eligibility

After the automatic and manual removal of duplicates, two review authors (MF and ZV) independently carried out the selection process first by titles, then by abstracts and full-texts. A third investigator (MI) resolved any arising controversies. EndNote X9 software (Clarivate Analytics, Philadelphia, PA, USA) was used for record management. Cohen’s kappa coefficient (κ) was calculated to measure the reliability of agreements during the selection process. κ values ≤ 0 is interpreted as no agreement, 0.01–0.20 as none to slight, 0.21–0.40 as fair, 0.41–0.60 as moderate, 0.61–0.80 as substantial, 0.81–1.00 as almost perfect and 1.00 as a perfect agreement [20].

Human studies (cohort, case-control and cross-sectional), both full-texts and conference abstracts, that reported on at least one of the pre-specified outcomes were eligible for inclusion. We only included studies in which three comparisons were reported: (1) Newly diagnosed CD patients vs. non-celiac control subjects, (2) CD patients at the time of the diagnosis vs. the same patients after at least a one-year GFD, (3) CD patients after at least a one-year GFD vs. non-celiac control subjects.

To be included, the diagnosis of CD had to be based on serological testing and intestinal biopsy or according to the recommendations of the pediatric guidelines [21,22]. Study populations with further selection (e.g., diabetic CD patients only, women only) were excluded. Studies recruiting patients from specific age groups were included. Study participants had to follow either a regular gluten-containing diet or a traditional GFD. Studies with further dietary modifications (e.g., a low-carb GFD, a GFD with vitamin B_12_ supplementation) were excluded. If a non-celiac control group was recruited, control subjects had to be declared to be healthy; otherwise, the study was excluded (the recruitment of, e.g., “other gastrointestinal patients” or “patients with negative endoscopy results” was not accepted).

### 2.3. Data Extraction

Data were extracted by two independent review authors (MF and ZV) using standardized data collection forms. Disagreements were resolved by a third investigator (MI).

We designed separate forms for each comparison of groups. The following parameters were collected: General characteristics of the study (authors, title, year of publication, study design), description of the population (sample size, age (years), gender, BW (kg or Z-score), body height (cm or Z-score), BMI (kg/m^2^ or Z-score)), diagnostic method of CD, follow-up period and the outcomes including FM (kg or % or Z-score), FFM (kg or % or Z-score), visceral fat area (cm^2^), total body water (% or Z-score), bone mineral content (BMC) (g or Z-score) and bone mineral density (BMD) (g/cm^2^ or Z-score). The year of publication and study sites were compared to identify overlapping populations.

### 2.4. Statistical Analysis

For meta-analytical calculations, we used means and standard deviations collected from the studies. In meta-analyses, pooled weighted mean differences (WMDs) with 95% confidence intervals (CIs) were calculated. The DerSimonian and Laird random-effects model was applied [23]. Cochrane’s Q and the I^2^ statistics were used to quantify heterogeneity. Forest plots were used to visually display the results of the meta-analysis. Due to the low number of studies, publication bias was not tested. The analysis was performed with STATA software version 15 (Stata, College Station, Texas).

### 2.5. Risk of Bias Assessment

The quality assessment of the studies included was performed by two independent review authors by applying the NIH Quality Assessment Tool for Observational Cohort and Cross-Sectional Studies (available at: https://www.nhlbi.nih.gov/health-topics/study-quality-assessment-tools, accessed on 22 July 2021). In studies comparing newly diagnosed CD patients to non-celiac controls, CD was defined as the exposure. In before–after studies reporting on CD patients, the GFD was defined as the exposure. In the case of cross-sectional studies, the domain on follow-up was inapplicable. For all studies, we had to omit the domain “Repeated exposure assessment” due to inadaptability.

## 3. Results

### 3.1. Search and Selection

**Figure 1:**
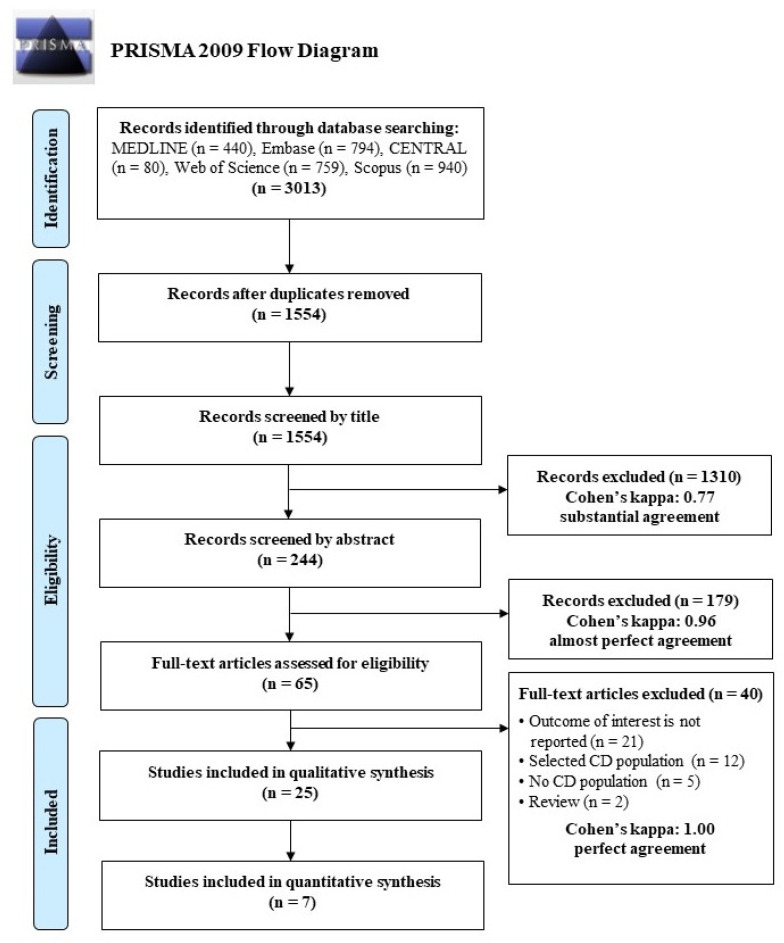
PRISMA flow chart describing the process of the study search and selection.

A total of 3013 records were identified from the electronic databases. After the automatic and manual removal of duplicates, 1554 records remained. After screening by title and abstracts, 65 studies were screened for eligibility; 25 of which were included in systematic review, seven of which (six full-text articles and one conference abstract) were eligible for meta-analysis. Studies recruiting only women were excluded [24,25]. A detailed description of the selection process with Cohen’s kappa coefficients is presented in Figure 1.

### 3.2. Characteristics of the Studies Included

In terms of the main outcomes, the studies applied three measurement modalities (that is, dual-energy X-ray absorptiometry (DXA), bioimpedance analysis (BIA), isotopic dilution (ID), skinfold thickness measurement (STM)); measurements were reported in various units.

Seven studies compared newly diagnosed CD patients to a non-celiac control group [10,15,26,27,28,29,30]. Due to the low number of studies with comparable measurement modalities and age groups, we could not perform meta-analysis on body composition-related parameters.

Nine studies compared CD patients at the time of the diagnosis to the same patients after at least a one-year GFD [10,26,30,31,32,33,34,35,36], three of which were included in the meta-analysis, all using DXA in adults [32,33,34]. Among the main outcomes, only the data on FM were reported in similar units.

Sixteen studies compared CD patients on a GFD to a non-celiac control group [9,10,11,13,14,15,16,26,27,37,38,39,40,41,42,43], only four of which could be included in the meta-analysis [11,13,26,37]. In terms of the main outcomes, only FM and FFM were reported in similar units. All CD patients followed a GFD for at least one year. These studies used DXA to assess body composition-related parameters in adults.

The characteristics of the studies included in systematic review and meta-analysis are shown in Table 1.

### 3.3. Results of Meta-Analysis

#### 3.3.1. CD Patients at Diagnosis vs. Same Patients on a GFD for at Least One Year

**Figure 2:**
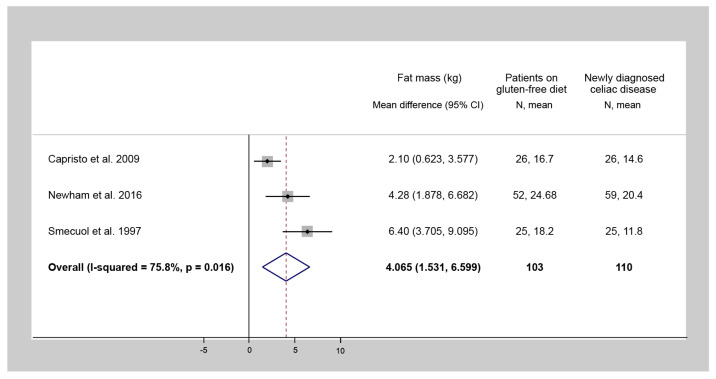
Forest plot of studies comparing fat mass of celiac disease patients after at least a one-year gluten-free diet to that of the same patients at diagnosis (a positive number indicates a gain in fat mass following a gluten-free diet). N: Number of patients.

Three studies evaluated FM of CD patients at the diagnosis and also after at least a one-year follow-up GFD. The one-year-long GFD treatment resulted in a statistically significant increase in FM (WMD = 4.1 kg, 95% CI = 1.5 to 6.6, I^2^ = 75.8%, *p* = 0.016) (Figure 2). The amount of data did not allow us to perform a meta-analysis on FFM; however, in most of the studies, the change during a GFD was not significant [10,26,32,33,34].

#### 3.3.2. CD Patients on a GFD for at Least One Year vs. Non-Celiac Control Subjects

**Figure 3:**
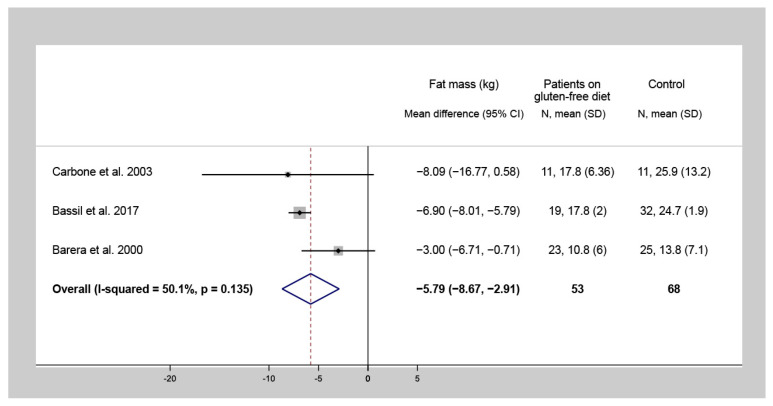
Forest plot of studies comparing fat mass of celiac disease patients on a gluten-free diet for at least one year to that of non-celiac control subjects (a negative number indicates a lower fat mass of the celiac population compared to controls). N: Number of patients.

**Figure 4:**
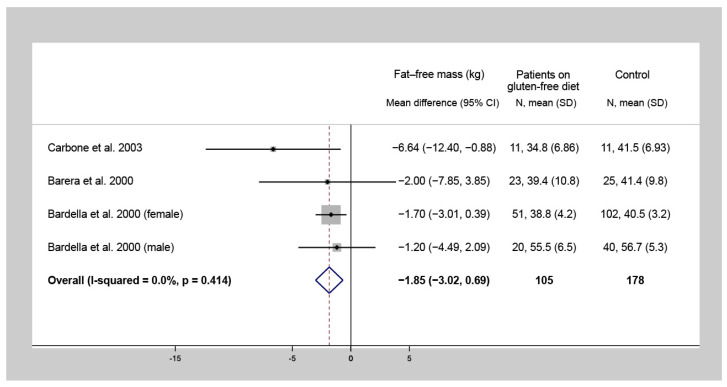
Forest plot of studies comparing fat-free mass of celiac disease patients on a gluten-free diet for at least one year to that of non-celiac control subjects (a negative number indicates a lower fat-free mass of the celiac population compared to controls). N: Number of patients.

Four studies investigated the difference of FM and FFM values between CD patients following a one-year-long GFD and control subjects. Among CD patients on a GFD for at least one year, lower FM (WMD = −5.8 kg, 95% CI = −8.7 to −2.9, I^2^ = 50.1%, *p* = 0.135) (Figure 3) and FFM (WMD = −1.9 kg, 95% CI = −3.0 to −0.7, I^2^ = 0.0%, *p* = 0.414) (Figure 4) were detected, compared to the control group.

### 3.4. Results of Systematic Review

Seven studies assessed newly diagnosed CD patients and non-celiac control subjects [10,15,26,27,28,29,30], four of which used DXA [26,27,29,30], two used BIA [15,28] and another one used ID [10] as a method for body composition analysis. Concerning the age groups, there were five studies including children [26,27,28,29,30] and another two including adults [10,15]. Since the studies used different methods for body composition analysis and recruited various age groups, meta-analysis could not be performed. However, in four studies, CD patients had significantly lower FM values [10,15,26,30], whereas in three studies, CD patients had significantly lower FFM values [10,15,30], compared to the control group. In the remaining study, there was no significant difference between the groups.

Nine studies assessed CD patients at the time of the diagnosis and the same patients after at least a one-year GFD [10,26,30,31,32,33,34,35,36], five of which used DXA [26,30,32,33,34], three used BIA [31,35,36] and one used ID [10] as a method for body composition analysis. Five studies recruited adults [10,32,33,34,35] and another four recruited children [26,30,31,36]. FM significantly increased in four studies [10,32,33,34] and significantly decreased in one study during a GFD [31]. FFM significantly increased in three studies [30,31,36] and significantly decreased in another study during a GFD [35]. In the remaining studies, FM and FFM did not change significantly during a GFD.

Sixteen studies assessed CD patients after at least a one-year GFD and non-celiac control subjects [9,10,11,13,14,15,16,26,27,37,38,39,40,41,42,43]. Eight used DXA [11,13,26,27,37,38,39,42], five used BIA [9,15,38,40,43], three used STM [14,16,41] and one used ID as a method for body composition analysis [10]. Ten studies included adults [10,11,13,14,15,26,37,39,40,41]; another seven included children [9,16,27,37,38,42,43]. One study used both DXA and BIA to assess body composition [38] and another one included both adults and children [37]. CD patients had significantly lower FM in five studies [10,11,13,14,15] and FFM in six studies [10,11,15,37,38,42] and significantly higher FFM in one study [14], compared to the control group. In one case, FFM among CD patients was significantly higher than that of controls [14]. In the other studies, a significant difference in FM and FFM values were not statistically significant.

The results of systematic review are summarized in Table 2.

Regarding the outcomes, only FM and FFM were published in all studies, the other outcomes are detailed in Table S2.

### 3.5. Risk of Bias Assessment

Regarding the comparability of the cohorts of patients, the studies were age- and/or gender-matched except for four studies matching by additional factors (height, social status, BMI and HLA-DQ) [10,27,39,40]. Concerning the assessment of outcomes, the measurement modalities have a valid methodology with an algorithm to estimate the ratio of body composition-related parameters, so that all studies carried a low risk of bias in this domain. In longitudinal studies, the follow-up period was judged to be sufficiently long; however, information about blinding and sample size justification was not reported in any study. In the domain “Statistical analyses”, the influence of confounding variables was not investigated in 17 studies. A summary of the risk of bias assessment is presented in the Supplementary Materials (Figure S1).

## 4. Discussion

In this study, we aimed to compare the body composition across CD patients before a GFD, CD patients after a one-year GFD and non-celiac control subjects.

While the difference in body composition between newly diagnosed CD patients and non-celiac control subjects could not be meta-analyzed due to the diversity in data, we observed that BW, BMI, FM, FFM, BMC and BMD values were lower in CD patients than in the non-celiac control group in most of the studies [10,15,26,27]. This can be attributed to malabsorption, the classical clinical presentation of CD. Consequently, the indicators of the nutritional status of newly diagnosed CD patients on a gluten-containing diet are usually worse than those observed in the average population.

Most of the studies that evaluated changes in body composition between CD patients at the time of the diagnosis and the same patients after at least a one-year follow-up period introduced a GFD as a BW, BMI and FM promoter [10,26,31,32,33,34]. Restored intestinal absorption and the unbalanced composition of a GFD, being rich in simple carbohydrates and saturated fats, resulted in weight gain [17,44]. A GFD induced BW gain; hence, BMI improvement can be considered optimal when the FFM ratio is higher than the FM; however, among most of the CD patients this is not the case. Our meta-analysis showed the same phenomenon, as we detected a significant increase in FM, but FFM mostly did not change during a one-year GFD. Albeit, after three or five years of diet, FFM tended to rise [33,34]. In contrast, the study by Rocco et al. assessed body composition at diagnosis and after at least 12 months of GFD and BMI plus FM did not change during the diet. However, the decreased FFM influenced the FM/FFM ratio unfavorably [35]. This means that BW and FM (thus fat deposits), may recover easily, contrasting FFM which is unable to normalize rapidly (≈one year) [34]. The disproportionate increase in FM is not desirable in CD patients who have normal body weight or are overweight at diagnosis.

Our meta-analysis on the changes of FM and FFM showed that these parameters do not reach the level of the non-celiac control population after a one-year GFD, corroborating previous findings [10,15]. The reason could be a poor dietary adherence, incomplete mucosal recovery, and lack of awareness about disease management.

While the majority of the studies included CD patients who had satisfactory compliance with a GFD, the degree of dietary adherence can range from partial to strict. Smecuol et al. and Wiech et al. reported that the improvement in body composition is more substantial in the case of a strict GFD [34,36]; however, in another study, the dietary adherence did not influence the nutritional status [11]. The heterogeneous nature of CD and the different national and cultural aspects in dietary habits could lead to further diversity.

In children, it is hard to distinguish between the effect of the diet and the normal growth on body composition, so that data of longitudinal, follow-up studies of different age groups (under 18 years) are barely comparable. For this reason, data on adults and children should be analyzed separately. Unfortunately, we could only perform meta-analysis relying on adult patients’ data.

Regarding other body composition parameters, BMC was lower in newly diagnosed CD patients than in controls [26]. After at least one year of GFD treatment, BMC tended to normalize [26] and, in the long-term (>one year), it was completely restored compared to control subjects [26,37].

BMD of patients who started a GFD in childhood was higher than that of patients first diagnosed in adulthood [11], indicating that the earlier the diagnosis the better the clinical outcomes [27,45,46].

Among the 25 studies, only one measured visceral fat area. The researchers observed a statistically not significant but measurable increase in the visceral fat area among treated CD patients, compared to controls. Moreover, the visceral fat area of 40% of CD patients on a GFD was above 100 cm^2^, indicating elevated risk for adverse metabolic alterations [40].

Four studies evaluated the effect of CD and GFD on total body water in children, yielding inconsistent findings [28,31,36,43].

Abnormal body composition of CD patients as well as changes in body composition during a GFD and the assessment of nutritional status at the diagnosis of CD and during regular follow-up visits are worth considering [11,26,27,38,47]. Information about body composition helps the early detection of malnutrition at diagnosis and supports the prevention of long-term complication of macro- and micronutrient deficiencies (e.g., short stature, osteoporosis). Several studies suggested that the earlier the diagnosis the better the nutrition education and consequently the body composition is expected to recover. However, a complete recovery more likely occurs in childhood [26,27,37] rather than in adulthood [11,13,15].

Previous findings supported the tendency that non-classical and silent forms of CD are becoming more frequent and the proportion of patients with a normal or high body weight at diagnosis is increasing rapidly [7,45,46,47]. The improvement of nutritional status was also observed both at the presentation of CD and after a GFD [8].

These data call attention to a need for management of the consequences of both the under- and overnourishment in the care of CD patients. A personalized diet and the promotion of a healthy diet and lifestyle are expected to trigger favorable trends in the changes of body composition.

### Strengths and Limitations

Since only narrative reviews are available [45,48,49,50], to our knowledge, this is the first meta-analysis in the literature that assessed the changes of body composition among CD patients with and without a GFD compared to non-celiac control subjects. Nevertheless, the extensive search and the stringent selection process are the main strengths of this study. We must refer to the fact that there are several limitations of this work as well.

First, the studies included were all observational, single-center studies because only such are available. Conference abstracts, which are usually not strictly peer-reviewed publications, were also included in the meta-analysis. The clinical manifestation of CD has not been precisely defined in previous studies, except for in two [10,34]. The next considerable limitation may be the variability in the follow-up period. The high heterogeneity in some analyses also could be highlighted. Due to the limited number of eligible studies with small sample sizes, publication bias could not be investigated. Our meta-analysis includes a relatively small number of studies, thus increasing the possibility of making a Type II error. Another important limitation of our meta-analysis is that, in some cases, the quality of the reported outcomes was rather poor. Conversion of medians to means could be a distortion factor in our results. We intended to perform subgroup analyses based on age (children and adults) and measurement modalities of body composition; however, there was no sufficient data to do so. Thus, we analyzed only studies using DXA in adults in meta-analysis, while all studies were included in the systematic review. Additionally, we had to omit a domain from the risk of bias assessment tool and there were domains which were not applicable in the majority of studies regarding different study designs. For this reason, the overall assessment could not be evaluated, thus cautious interpretation of the results is required.

## 5. Conclusions

The body composition of CD patients differs from that of the non-celiac population. A GFD was associated with a substantial gain in FM and a modest increase in FFM; however, even after a longstanding GFD, these parameters did not reach the optimal.

### 5.1. Implications for Clinical Practice

Current CD guidelines do not recommend the baseline and follow-up body composition assessment. The findings of our review suggest that follow-up of the nutritional status in addition to body composition measurements and personalized dietary counseling are important to prevent the long-term consequences of malnutrition and disproportionate weight gain.

### 5.2. Implications for Research

Prospective, well-designed studies recruiting a sufficient number of CD patients investigating body composition and its changes during a GFD are awaited.

## Figures and Tables

**Table 1:** Characteristics of the studies included in systematic review and meta-analysis.

Publication	Age Group	Matching	Body Composition Analysis	No. of Patients	Groups	Length of Gluten-Free Diet (Year)	Outcome									
							Body Weight		Body Mass Index		Fat mass			Fat-Free Mass		
							kg	Other Unit	kg/m^2^	Other Unit	kg	%	Other Unit	kg	%	Other Unit
	**Newly diagnosed celiac patients vs. non-celiac control subjects**															
Capristo et al., 2000 [10]	adults	age, height	ID	39	newly diagnosed	N/A	58.2 ± 6.7				11.6 ± 4.0	20.1 ± 6.8		46.6 ± 7.5	79.9 ± 6.8	
				63	control		67.0 ± 6.6				16.9 ± 3.0	25.4 ± 4.0		50.2 ± 6.2	74.8 ± 4.2	
Capristo et al., 1997 [15]	adults	age, gender	BIA	16	newly diagnosed	N/A	54.9 (41.0–72.0)		19.9 (16.6–28.8)		13.4 (6.2–30.7)	24.2 (11.1–42.6)		41.8 (30.4–50.9)	76.6 (57.4–89.1)	
				20	control		66.4 (57.0–76.0)		23.6 (19.4–26.0)		17.4 (9.9–24.2)	26.7 (14.2–35.9)		49.6 (36.7–60.9)	74.2 (64.1–85.8)	
Barera et al., 2000 [26]	children	age, gender	DXA	29	newly diagnosed	N/A	28.3 ± 11.0		16.4 ± 3.8		4.6 ± 3.5	17.4 ± 8.3		21.4 ± 8.4		
				29	control		34.5 ± 14.1		18.1 ± 2.8		7.5 ± 4.9	23.7 ± 8.4		23.4 ± 10.3		
Björck et al., 2017 [27]	children	age, gender, HLA-DQ	DXA	71	newly diagnosed	N/A	37.2 ± 8.2		17.9 ± 3.0		11.0 ± 5.6			24.8 ± 3.4		
				142	control		38.6 ± 8.2		18.5 ± 3.0		11.7 ± 5.5			25.5 ± 3.7		
Aurangzeb et al., 2010 [28]	children	age, gender	BIA	25	newly diagnosed	N/A		for age percentile: 45 ± 29.9		percentile: 50.5 ± 31.4			Rush equation: 6.3 ± 7.3			Rush equation: 23.4 ± 11.0
				25	control			for age percentile: 46.6 ± 31.1		percentile: 48.2 ± 32.2			Rush equation: 6.2 ± 6.5			Rush equation: 23.5 ± 11.0
Rätsch et al., 2001 [29]	children	-	DXA	65	newly diagnosed	N/A		Z-score: −2.2 ± 0.1				25.8 ±12.4			74.9 ± 12.3	
				71	control			Z-score: −2.1 ± 0.2				24.9 ± 9.5			75.9 ± 9.4	
Gallardo et al., 2008 [30] *	children	age, gender	DXA	29	newly diagnosed	N/A	significantly lower in CD than in control subjects		significantly lower in CD than in control subjects		significantly lower in CD than in control subjects			significantly lower in CD than in control subjects		
				32	control											
	**Celiac patients at diagnosis vs. same patients after at least a one-year gluten-free diet**															
Capristo et al., 2000 [10]	adults	N/A	ID	39	before a GFD		58.2 ± 6.7				11.6 ± 4.0	20.1 ± 6.8		46.6 ± 7.5	79.9 ± 6.8	
					on a GFD	1.0 ± 0.0	60.9 ± 6.2				13.8 ± 3.7	22.9 ± 6.2		47.1 ± 7.0	77.2 ± 6.1	
Barera et al., 2000 [26]	children	N/A	DXA	20	before a GFD		30.3 ± 11.5		16.7 ± 4.5		5.0 ± 4.1	16.9 ± 8.9		23.2 ± 8.2		
					on a GFD	1.02 ± 0.2	34.7 ± 12.3		17.3 ± 3.1		6.2 ± 4.2	19.4 ± 8.0		26.0 ± 9.3		
Gallardo et al., 2008 [30] *	children	N/A	DXA	10	before a GFD		significantly increased after a GFD		significantly increased after a GFD		did not change significantly after a GFD			significantly increased after a GFD		
					on a GFD	≥1										
Suárez-González et al., 2020 [31]	children	N/A	BIA	72	before a GFD			Z-score: 0.2 ± 1.1				20.4 ± 9.3			79.5 ± 9.4	
					on a GFD	2 (0.7–11.5)		Z-score: 0.1 ± 0.1				16.9 ± 8.6			83.1 ± 8.4	
Capristo et al., 2009 [32]	adults	N/A	DXA	26	before a GFD		60.3 ± 3.8		22.1 ± 1.2		14.6 ± 2.6			45.7 ± 4		
					on a GFD	1.2 ± 0.1	63.0 ± 3.5		23.1 ± 1.3		16.7 ± 2.7			46.2 ± 3.9		
Newnham et al., 2016 [33]	adults	N/A	DXA	52	before a GFD		68.1 ± 12.3		24.1 ± 3.5		20.4 ± 5.9	31.4 ± 8.3		46.5 ± 8.5		
					on a GFD	1	71.1 ± 14.4		25.0 ± 4.2		24.7 ± 10.3	34 ± 8.8		46.4 ± 9.4		
Smecuol et al., 1997 [34]	adults	N/A	DXA	25	before a GFD		48.6 ± 2.2		19.5 ± 0.7		11.8 ± 1.5			33.4 ± 1.4		
					on a GFD	3.1 (2.2–4.1)	55.7 ± 2.3		22.2 ± 0.7		18.2 ± 1.7			35.3 ± 1		
Rocco et al., 2014 [35] *	adults	N/A	BIA	15	before a GFD						19.9			50.2		
					on a GFD	≥1			did not change significantly after a GFD		did not change significantly after a GFD			significantly decreased after a GFD		
Wiech et al., 2018 [36]	children	N/A	BIA	22	before a GFD		32.4 ± 15.7		16.8 ± 2.8		7.2 ± 4.6	22.1 ± 6.5		25.2 ± 12.2	78.0 ± 6.5	
					on a GFD	1.4	36.0 ± 14.1		17.1 ± 2.1		7.4 ± 3.8	21.2 ± 6.9		28.6 ± 11.9	78.8 ± 6.9	
	**Celiac patients on a gluten-free diet for at least one year vs. non-celiac control subjects**															
Tsiountsioura et al., 2014 [9]	children	-	BIA	26	on a GFD			Z-score: −0.0 ± 1.2		Z-score: 0.1 ± 1.1			Z-score: 0.3 ± 1.2			Z-score: 0 ± 1.0
				54	control			Z-score: 0.2 ± 1.1		Z-score: 0.4 ± 1.5			Z-score: 0.4 ± 1.3			Z-score: 0.2 ± 1.2
Capristo et al., 2000 [10]	adults	age, height	ID	39	on a GFD	1.0 ± 0.0	61.4 ± 5.7				13.3 ± 2.7	21.9 ± 3.5		48.1 ± 4.9	78.2 ± 3.4	
				63	control		67.0 ± 6.6				16.9 ± 3.0	25.4 ± 4.0		50.2 ± 6.2	74.8 ± 4.2	
Bardella et al., 2000 [11]	adults	age, gender	DXA	71	on a GFD	≥2	59.4 ± 10.8		21.2 ± 2.8			20.4 ± 6.4		43.5 ± 9.0		
				142	control		62.8 ± 10.8		22.7 ± 4.0			24.5 ± 7.0		45.1 ± 8.3		
Bassil et al., 2017 [13] *	adults	age, gender	DXA	19	on a GFD		63.3 ± 3.0		22.2 ± 0.8		17.8 ± 2.0					
				32	control		25.7 ± 0.7		25.7 ± 0.7		24.7 ± 1.9					
Bodé et al., 1991 [14]	adults	age	STM	22	on a GFD	8.1 ± 6.0			significantly lower in CD than in control subjects		significantly lower in CD than in control subjects			significantly higher in CD than in control subjects		
				-	control											
Capristo et al., 1997 [15]	adults	age, gender	BIA	18	on a GFD	3.7 (1–6.3)	55.6 (40.0–66.0)		20.2 (14.9–25.8)		11.7 (5.3–24.4)	20.9 (9.7–37.0)		42.9 (31.5–56.4)	78.7 (63.0–90.3)	
				20	control		66.4 (57.0–76.0)		23.6 (19.4–26.0)		17.4 (9.88–24.2)	26.7 (14.2–35.9)		49.6 (36.7–60.9)	74.2 (64.1–85.8)	
Ballestero-Fernández et al., 2019 [16]	children	age, gender	STM	70	on a GFD	≥1	34.8 ± 4.9		17.2 ± 0.7			17 ± 2.0				
				67	control		38 ± 4.8		18.5 ± 1.2			17.7 ± 2.1				
Barera et al., 2000 [26]	adults	age, gender	DXA	23	on a GFD	10.6 ± 4.5	54.2 ± 10.9		21.4 ± 3.2		10.8 ± 6	22.1 ± 10.3		39.4 ± 10.8		
				25	control		58.5 ± 11.8		20.9 ± 2.7		13.8 ± 7.1	24.4 ± 10.2		41.4 ± 9.8		
Björck et al., 2017 [27]	children	age, gender, HLA-DQ	DXA	30	on a GFD	6.9 ± 1.1	41.6 ± 10.2		18.8 ± 3.4		13.1 ± 6.4			27 ± 4.8		
				60	control		41.7 ± 10.1		18.4 ± 3.4		12.7 ± 6.3			27.5 ± 4.7		
Carbone et al., 2003 [37]	adults	age, gender	DXA	11	on a GFD	0.8–13	55.3 ± 10.4				17.8 ± 6.4			34.8 ± 6.9		
				11	control		69.6 ± 13.6				25.9 ± 13.2			41.5 ± 6.9		
	children		DXA	48	on a GFD	0.8–13	50.5 ± 11.8				15.6 ± 7.0			32.5 ± 8.5		
				30	control		62.2 ± 12				12.9 ± 8.8			46.3 ± 12.5		
De Lorenzo et al., 1999 [38]	children	age, gender	DXA	43	on a GFD	1 ± 0.3	48.8 ± 11.4		19.8 ± 3.1		15 ± 6.8			31.6 ± 8.1		
				30	control		62.2 ± 12.0		21.6 ± 3.0		12.9 ± 8.8			46.3 ± 12.5		
			BIA	43	on a GFD	1 ± 0.3	48.8 ± 11.4		19.8 ± 3.1		19.3 ± 8.8			32.4 ± 8.1		
				30	control		62.2 ± 12.0		21.6 ± 3.0		18.9 ± 9.4			45.4 ± 10.2		
Barone et al., 2015 [39]	adults	age, gender, social status	DXA	39	on a GFD	2.2 ± 0.9						29.9 ± 7.8				
				39	control							29.8 ± 7.8				
Nunes-Silva et al., 2017 [40]	adults	age, gender BMI	BIA	15	on a GFD	6–12			22.9 ± 3.6			37.3 ± 4.4				
				15	control				23.1 ± 2.7			32.7 ± 10.6				
Ballestero-Fernández et al., 2021 [41]	adults	age, gender	STM	64	on a GFD	≥1	66 ± 5.4		22.8 ± 1.6			30.5 ± 3.6				
				74	control		64.3 ± 4.1		23.5 ± 1.6			29.8 ± 3.2				
Nestares et al., 2021 [42]	children	age, gender	DXA	41	on a GFD	≥1.5	significantly lower in CD than in control subjects		no significant difference between CD and control subjects		no significant difference between CD and control subjects			significantly lower in CD than in control subjects		
				40	control											
Silva et al., 2014 [43]	children	age, gender	BIA	31	on a GFD	≥1	no significant difference between CD and control subjects		no significant difference between CD and control subjects		no significant difference between CD and control subjects			no significant difference between CD and control subjects		
				31	control											

* presented: Conference abstracts; studies with underline are included in meta-analyses; BIA: Bioimpedance analysis; DXA: Dual-energy X-ray absorptiometry; ID: Isotopic dilution; STM: Skinfold thickness measurement; GFD: Gluten-free diet; N/A: Not applicable; values are reported in mean and standard deviation: $\bar{x}$ ± SD; or mean and range: $\bar{x}$(range).

**Table 2:** Results of systematic review.

**Newly diagnosed celiac patients vs. non-celiac control subjects**						
**Publication**	**Age group**	**Body composition analysis**	**Outcome (reference: control group)**			
			**Body weight**	**Body mass index**	**Fat mass**	**Fat-free mass**
Capristo et al., 2000 [10]	adults	ID	↓	N/A	↓	↓
Capristo et al., 1997 [15]	adults	BIA	↓	↓	↓	↓
Barera et al., 2000 [26]	children	DXA	↓	-	↓	-
Björck et al., 2017 [27]	children	DXA	-	-	-	-
Aurangzeb et al., 2010 [28]	children	BIA	-	-	-	-
Rätsch et al., 2001 [29]	children	DXA	-	N/A	-	-
Gallardo et al., 2008 [30] *	children	DXA	↓	↓	↓	↓
**Celiac patients at diagnosis vs. same patients after at least a one-year gluten-free diet**						
**Publication**	**Age group**	**Body composition analysis**	**Outcome (reference: celiac patients after at least a 1-year gluten-free diet)**			
			**Body weight**	**Body mass index**	**Fat mass**	**Fat-free mass**
Capristo et al., 2000 [10]	adults	ID	↑	N/A	↑	-
Barera et al., 2000 [26]	children	DXA	-	-	-	-
Gallardo et al., 2008 [30] *	children	DXA	↑	↑	-	↑
Suárez-González et al., 2020 [31]	children	BIA	N/A	-	↓	↑
Capristo et al., 2009 [32]	adults	DXA	↑	↑	↑	-
Newnham et al., 2016 [33]	adults	DXA	↑	↑	↑	-
Smecuol et al., 1997 [34]	adults	DXA	↑	↑	↑	-
Rocco et al., 2014 [35] *	adults	BIA	-	-	-	↓
Wiech et al., 2018 [36]	children	BIA	↑	↑	-	↑
**Celiac patients on a gluten-free diet for at least one year vs. non-celiac control subjects**						
**Publication**	**Age group**	**Body composition analysis**	**Outcome (reference: control group)**			
			**Body weight**	**Body mass index**	**Fat mass**	**Fat-free mass**
Tsiountsioura et al., 2014 [9]	children	BIA	-	-	-	-
Capristo et al., 2000 [10]	adults	ID	↓	N/A	↓	↓
Bardella et al., 2000 [11]	adults	DXA	↓	↓	↓	↓
Bassil et al., 2017 [13] *	adults	DXA	↓	↓	↓	N/A
Bodé et al., 1991 [14]	adults	STM	N/A	↓	↓	↑
Capristo et al., 1997 [15]	adults	BIA	↓	↓	↓	↓
Ballestero-Fernández et al., 2019 [16]	children	STM	-	-	-	N/A
Barera et al., 2000 [26]	adults	DXA	-	-	-	-
Björck et al., 2017 [27]	children	DXA	-	-	-	-
Carbone et al., 2003 [37]	children	DXA	↓	N/A	-	↓
	adults		↓	N/A	-	↓
De Lorenzo et al., 1999 [38]	children	DXA	↓	↓	-	↓
		BIA	↓	↓	-	↓
Barone et al., 2015 [39]	adults	DXA	N/A	N/A	-	-
Nunes-Silva et al., 2017 [40]	adults	BIA	N/A	-	-	N/A
Ballestero-Fernández et al., 2021 [41]	adults	STM	-	-	-	N/A
Nestares et al., 2021 [42]	children	DXA	↓	-	-	↓
Silva et al., 2014 [43]	children	BIA	-	-	-	-

↑: Significantly higher; ↓: Significantly lower; -: Not significant; N/A: No data available; * Conference abstract; BIA: Bioelectrical impedance analysis; DXA: Dual-energy X-ray absorptiometry; ID: Isotopic dilution; STM: Skinfold thickness measurement.

## Data Availability

The data presented in this study are available within the article and the Supplementary Materials. The raw data are available on request from the corresponding author.

## Supplementary Materials

The following are available online at https://www.mdpi.com/article/10.3390/nu13092947/s1, Table S1: PRISMA 2020 checklist, Figure S1: Risk of bias assessment, Table S2: Outcome not subjected to meta-analyses.
